# Patient Perspectives on the Cultural Competence of US Health Care Professionals

**DOI:** 10.1001/jamanetworkopen.2019.16105

**Published:** 2019-11-27

**Authors:** Lynn A. Blewett, Rachel R. Hardeman, Robert Hest, Tyler N. A. Winkelman

**Affiliations:** 1 Division of Health Policy and Management, University of Minnesota School of Public Health, Minneapolis; 2 State Health Access Data Assistance Center, Division of Health Policy and Management, University of Minnesota School of Public Health, Minneapolis; 3 Hennepin Healthcare Research Institute, Minneapolis, Minnesota; 4 Division of General Internal Medicine, Department of Medicine, Hennepin Healthcare, Minneapolis, Minnesota

## Abstract

This survey study uses data from the 2017 National Health Interview Survey to examine patients’ perspectives on the cultural competence of US health care professionals.

## Introduction

Racial disparities in access to health care and health outcomes are well documented.^1^ One approach to reducing disparities has been to increase the cultural competency of health care professionals.^2^ Although the cultural competency of US health care professionals has received considerable attention, patients’ views regarding the competency of their health care professionals have not been fully examined.^3^ To fill this gap, the Office of the Assistant Secretary for Health’s Office of Minority Health and Health Equity sponsored 5 cultural competency questions on the 2017 National Health Interview Survey (NHIS).^4^ We used these questions to examine patients’ perspectives on the cultural competence of US health care professionals.

## Methods

This survey study used data from the 2017 NHIS, a cross-sectional, nationally representative household survey with a sample of 78 543 participants accessed through the IPUMS Health Surveys website.^4^ The NHIS has collected data on US health behaviors, health status, and access to health care for more than 50 years. A total of 22 864 patients aged 18 years and older who had seen a physician or health care professional in the past year were asked 5 questions about their visit. The total household response rate was 66.5%, consistent with American Association for Public Opinion Research (AAPOR) reporting guidelines for household surveys.^5^ We used logistic regression models to generate unweighted and weighted response estimates by race/ethnicity, insurance status, poverty level, and education adjusted for age, sex, and the presence of 1 or more chronic conditions. We conducted significance testing using 2-sided *t* tests and considered *P* < .05 to be statistically significant. Data were analyzed with Stata statistical software version 16.0 (StataCorp) to account for the complex design of the NHIS. Data from the NHIS are publicly available with no individual identifiers, so analyses are exempt from institutional review board review according to the US Department of Health and Human Services Office of Human Research Protection.^6^

## Results

Among respondents eligible for the 5 survey questions, the mean (SD) age was 48.8 (18.3) years, 54.4% were female, and 64.4% identified as white (non-Hispanic). Table 1 provides unadjusted responses to the 5 NHIS questions. Nearly all respondents reported being treated with respect and receiving easy-to-understand information by health care professionals always (18 451 [81.5%]) or most of the time (3411 [15.1%]). Fewer respondents reported that health care professionals asked their opinions or beliefs about care always (7921 [35.6%]) or most of the time (5173 [22.9%]). More than one-third said that it was very important (4018 [19.2%]) or somewhat important (4038 [18.4%]) that their health care professionals understand or share their culture, whereas more than one-half of the respondents (12 014 [51.5%]) said that sharing one’s culture was not important at all.

**Table 1.** Unadjusted Responses to 2017 National Health Interview Survey Cultural Competence Questions^a^

Response	Participants, Unweighted No. (Weighted %)
How often treated with respect by providers^b^	
Always	18 451 (81.5)
Most of the time	3411 (15.1)
Some of the time	599 (2.7)
None of the time	148 (0.7)
How often providers give easy-to-understand information	
Always	15 106 (67.0)
Most of the time	5693 (24.8)
Some of the time	1372 (6.3)
None of the time	404 (1.9)
How often providers ask opinions or beliefs about care	
Always	7921 (35.6)
Most of the time	5173 (22.9)
Some of the time	4410 (19.6)
None of the time	4910 (21.9)
How important is it for providers to understand or share culture^c^	
Very	4018 (19.2)
Somewhat	4038 (18.4)
Slightly	2470 (10.9)
Not at all	12 014 (51.5)
How often able to see providers who share culture^c^^,^^d^	
Always	4157 (38.0)
Most of the time	3410 (32.2)
Some of the time	2086 (21.7)
None of the time	749 (8.2)

^a^ Sample includes 22 864 adults (aged ≥18 years) who saw a physician or other health professional in the past 12 months.

^b^ “Providers” includes physicians, clinicians, and other health care professionals.

^c^ Culture is defined as race, ethnicity, gender, religion, beliefs, or native language.

^d^ Among respondents who responded at least “slightly important” when asked how important it is for health care professionals to understand or share culture.

Table 2 provides weighted responses adjusted by sociodemographic characteristics. Individuals who identified as black non-Hispanic (2469 [95.1%; 95% CI, 93.9%-96.3%]; *P* < .001), Hispanic (2485 [95.6%; 95% CI, 94.5%-96.7%]; *P* < .001), or other race/ethnicity (1379 [95.4%; 95% CI, 94.0%-96.7%]; *P* = .01) were significantly less likely, compared with non-Hispanic white participants (16 276 [97.2%; 95% CI, 96.9%-97.6%]), to report that health care professionals treated them with respect most of the time or always. Significantly fewer uninsured (1330 [94.0%; 92.2%-95.8%]; *P* = .003) and low-income (6518 [94.4%; 95% CI, 93.6%-95.2%]; *P* < .001) individuals responded that they were treated with respect most of the time or always compared with their insured (21 225 [96.8%; 95% CI, 96.5%-97.1%]) and higher-income (15 086 [97.4%; 95% CI, 97.0%-97.7%]) peers. (In calendar year 2016, 200% of the federal poverty level was $23 760 with 1 person in the household and $40 320 for a family of 3.)

**Table 2.** Adjusted Responses to 2017 National Health Interview Survey Cultural Competence Questions by Sociodemographic Characteristics^a^

Response	Participants, Weighted % (Unweighted No.)								
	Race/Ethnicity				Insurance Status		Poverty Level		Total
	Non-Hispanic		Hispanic, Any Race^b^	All Others^b^	Insured	Uninsured	At or Above 200% FPL	Below 200% FPL	
	White	Black^b^							
How often treated with respect by providers (most of the time or always)^c^	97.2 (16 276)	95.1 (2469)^d^	95.6 (2485)^e^	95.4 (1379)^f^	96.8 (21 225)	94.0 (1330)^g^	97.4 (15 086)	94.4 (6518)^d^	96.6 (22 609)
How often providers give easy to understand information (most of the time or always)	93.0 (16 258)	89.9 (2462)^d^	89.2 (2480)^d^	88.9 (1375)^d^	92.2 (21 191)	86.9 (1330)^d^	93.3 (15 072)	88.1 (6504)^d^	91.8 (22 575)
How often providers ask opinions or beliefs about care (most of the time or always)	57.0 (16 130)	60.1 (2453)^h^	61.2 (2468)^i^	64.0 (1363)^d^	58.4 (21 044)	60.5 (1318)	57.8 (14 980)	60.0 (6456)^h^	58.5 (22 414)
How important is it for providers to understand or share culture (somewhat or very important)^j^	30.2 (16 232)	52.3 (2460)^d^	52.9 (2477)^d^	51.4 (1371)^d^	36.8 (21 157)	49.0 (1329)^d^	34.0 (15 056)	46.8 (6491)^d^	37.6 (22 540)
How often able to see providers who share culture (most of the time or always)^j^^,^^k^	78.0 (6646)	59.7 (1447)^d^	60.2 (1484)^d^	59.1 (825)^d^	71.1 (9638)	61.0 (734)^d^	71.6 (6538)	67.1 (3395)^d^	70.2 (10 402)

Abbreviation: FPL, federal poverty level (in calendar year 2016, 200% of the federal poverty level was $23 760 with 1 person in the household and $40 320 for a family of 3).

^a^ Sample includes 22 864 adults (aged ≥18 years) who saw a physician or other health professional in the past 12 months. Weighted percentages are adjusted by age, sex, and chronic condition status.

^b^ Comparison group is white, non-Hispanic.

^c^ “Providers” includes physicians, clinicians, and other health care professionals.

^d^ *P* < .001.

^e^ *P* = .008.

^f^ *P* = .01.

^g^ *P* = .003.

^h^ *P* = .03.

^i^ *P* = .005.

^j^ Culture is defined as race, ethnicity, gender, religion, beliefs, or native language.

^k^ Among respondents who responded at least “slightly important” when asked how important it is for health care professionals to understand or share culture.

Significantly more nonwhite (black, 2460 [52.3%; 95% CI, 49.6%-55.1%]; Hispanic, 2477 [52.9%; 95% CI, 50.3%-55.6%]; other race/ethnicity, 1371 [51.4%; 95% CI, 47.4%-55.3%]; *P* < .001 for all), uninsured (1329 [49.0%; 95% CI, 45.1%-52.9%]; *P* < .001), and low-income (6491 [46.8%; 95% CI, 44.8%-48.8%]; *P* < .001) individuals responded that it was somewhat or very important for health care professionals to understand or share their culture, compared with non-Hispanic white participants (16 232 [30.2%; 95% CI, 29.0%-31.3%]), those with insurance (21 157 [36.8%; 95% CI, 35.6%-37.9%]), and those with higher income (15 056 [34.0%; 95% CI, 32.7%-35.3%]). These groups were also less likely to see health care professionals who share their culture most of the time or always (white, 15 056 [78.0%; 95% CI, 76.6%-79.4%] vs black, 1447 [59.7%; 95% CI, 56.4%-63.1%], Hispanic, 1484 [60.2%; 95% CI, 56.8%-63.6%], and other race/ethnicity, 825 [59.1%; 95% CI, 54.7%-63.5%], *P* < .001 for all; insured, 9638 [71.1%; 95% CI, 69.8%-72.5%] vs uninsured, 734 [61.0%; 95% CI, 56.9%-65.0%], *P* < .001; and at or above poverty level, 6538 [71.6%; 95% CI, 70.0%-73.2%] vs below poverty level, 3395 [67.1%; 95% CI, 64.9%-69.3%], *P* < .001).

## Discussion

Using 5 new cultural competency questions in the NHIS, we found that most patients reported that they were treated with respect and received easy-to-understand information from their health care professionals. Importantly, we also found that nonwhite, low-income, and uninsured patients were less likely to report being treated with respect and more likely to view health care professionals’ knowledge of culture as important, which highlights deficiencies in providing access to culturally appropriate care for these populations. Our results are limited by the cross-sectional nature of the data and the sample’s restriction to individuals who were seen by a health care professional in the past year. Medical schools should consider improving the pipeline of diverse health care professionals and increasing efforts to eliminate structural racism that persists in the health care delivery system.^7^
